# *Lactobacillus rhamnosus* CNCM I-3690 decreases subjective academic stress in healthy adults: a randomized placebo-controlled trial

**DOI:** 10.1080/19490976.2022.2031695

**Published:** 2022-02-07

**Authors:** Lucas Wauters, Luka Van Oudenhove, Alison Accarie, Karlien Geboers, Hannelore Geysen, Joran Toth, Anja Luypaerts, Kristin Verbeke, Tamara Smokvina, Jeroen Raes, Jan Tack, Tim Vanuytsel

**Affiliations:** a Department of Gastroenterology and Hepatology, University Hospitals Leuven, Leuven, Belgium; b Translational Research in Gastrointestinal Disorders Ku Leuven, Leuven, Belgium; c Danone Nutricia Research, Palaiseau, France; d Vib Center for Microbiology, Leuven, Belgium; e Department of Microbiology and Immunology, Rega Institute, Leuven, Belgium

**Keywords:** *Lactobacillus rhamnosus*, probiotic, permeability, stress, gut-brain interaction

## Abstract

Psychological stress negatively affects the intestinal barrier function in animals and humans. We aimed to study the effect of *Lactobacillus rhamnosus* CNCM I-3690 on intestinal permeability and stress-markers during public speech. Healthy students were randomized to *L. rhamnosus*-containing (test) or acidified (placebo) milk consumed twice daily for 4 weeks, with 46 subjects per treatment group. Small intestinal permeability was quantified by a 2 h urinary lactulose–mannitol ratio (LMR, primary outcome), fractional excretion of lactulose (FEL) and mannitol (FEM). Salivary cortisol, State-Trait Anxiety Inventory (STAI) and Perceived Stress scores (PSS) were collected. No between-treatment differences were found for LMR (p = .71), FEL or FEM. Within-treatment analyses showed similar LMR and FEL but a stress-induced increase of FEM with the placebo (p < .05) but not test product. Despite a similar increase in salivary cortisol, the stress-induced increase in STAI was significantly lower with the test product vs. placebo (p = .01). Moreover, a stress-preventative effect of the probiotic was found for PSS and more pronounced in subjects with high stress-induced cortisol (p = .01). While increased FEM was mediated by salivary cortisol levels, the effect of the test product on subjective stress was not mediated by changes in FEM. No serious adverse events occurred. In conclusion, we demonstrated that *L. rhamnosus* CNCM I-3690 prevented stress-induced hyperpermeability to mannitol. Subjective but not objective stress-markers were reduced with *L. rhamnosus* vs. placebo, suggesting anxiolytic effects, which were independent of barrier stabilization and attractive for the reduction of stress in both health and disease. **Clinicaltrials.gov**, number NCT03408691.

## Introduction

The study of the interaction between psychological stress and gastrointestinal (GI) function is a complex and developing field. The bidirectional communication between the gut and the brain or gut-brain axis has been considered a pivotal player in the pathogenesis of both irritable bowel syndrome (IBS) and inflammatory bowel diseases (IBD).^1,2^ However, the exact mechanisms through which changes in the gut alter brain functioning, feelings, and behavior remain unclear. Gut microbes may play an important role as germ-free mice showed exaggerated responses to stress compared to specific pathogen-free mice, which were reversible through re-colonization with *Bifidobacterium infantis*.^3^ Probiotics are live micro-organisms that, when ingested in adequate amounts, exert a health benefit on the host and offer the opportunity to modulate the gut microbiota and thus central nervous function.^4^ Indeed, recent systematic reviews suggested that some *Lactobacillu*s and *Bifidobacterium* strains may reduce anxiety and *L. rhamnosus* was identified as a potential anxiolytic species based on preclinical data.^5,6^ *L. rhamnosus* JB-1, formerly referred to as *L. reuteri*, reduced depressive-like behavior in mice,^7,8^ which was abolished after vagotomy.^7^ However, these promising preclinical findings were not reproduced with *L. rhamnosus* JB-1 in healthy humans after a socially evaluated cold pressor test.^9^

Interestingly, preclinical studies have identified increased intestinal permeability as the potential link between psychological stress and mucosal immune activation via enhanced passage of antigens,^10^ with improvement after probiotics.^11,12^ Increased intestinal permeability has been demonstrated in IBS and IBD with mucosal eosinophil and/or mast cell activation.^13,14^ We showed that a social-evaluative stressor (public speech) and intravenous administration of CRH increased small intestinal permeability quantified by the urinary lactulose-to-mannitol ratio (LMR) in healthy students. This effect was abolished after pre-treatment with the mast cell stabilizer disodium-cromoglycate (DSCG).^15^ Recent studies demonstrated that both commensal and lactic acid-producing bacteria can enhance mucosal barrier integrity in mice inflammation models, with a similar protective effect of *L. rhamnosus* CNCM I-3690 compared to *Faecalibacterium prausnitzii* A2–165.^16^ Furthermore, anti-inflammatory effects were reported for *L. rhamnosus* in these models,^17,18^ making this an important candidate for further human research. However, no studies have investigated the potential role of bacteria in general or *L. rhamnosus* in particular for increased permeability during psychological stress in humans.

We therefore conducted a randomized, double-blind placebo-controlled trial to study the effect of a probiotic on stress-induced hyperpermeability (ProSPer) with *L. rhamnosus* CNCM I-3690 in healthy adults undergoing an oral thesis defense. We hypothesized that increased permeability during stress would be attenuated by *L. rhamnosus* compared to a placebo intervention. In addition, objective and subjective markers of stress were measured to assess the effect of *L. rhamnosus* on the biological and behavioral stress-response.

## Results

### Participants and adherence

**Figure 1.**
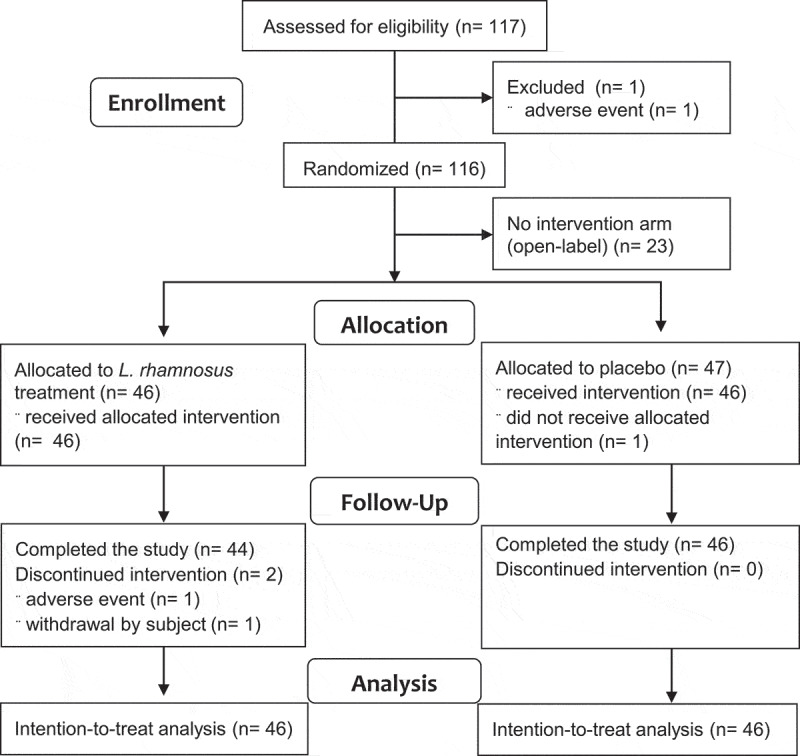
Study flowchart.

From January to July 2018, 117 subjects were included and 116 were randomized after exclusion of 1 subject during run-in, of which 93 to the intervention arms (Figure 1). A total of 44 of the 46 subjects with the test product (96%) and 46 of the 47 subjects with control product (98%) completed the study. There were no differences in the baseline characteristics including demographics and psychological screening questionnaires (Table 1). Proportions of study degree (bachelor or master) or topic (biomedical sciences, pharmacological sciences, etc.) were similar in the test vs. control product group, and all subjects fulfilled the pre-specified compliance criteria (80–120%) (see **Supplementary material**).

**Table 1.** Baseline characteristics

**Group**	***L. rhamnosus* product (n = 46)**	**Placebo product** **(n = 46)**	**p-value**
**Demographic**: • Age (years) • Female (%) • BMI	23.23 ± 0.32 26 (56) 21.97 ± 0.27	22.84 ± 0.28 28 (61) 21.56 ± 0.35	0.35 0.67 0.35
**Screening questionnaire**: • GAD-7 • PHQ-9	0.91 ± 0.19 1.20 ± 0.17	1.24 ± 0.19 1.41 ± 0.22	0.29 0.48
**In vivo permeability**: • LMR • FEL (%) • FEM (%)	0.03 ± 0.003 0.12 ± 0.008 9.54 ± 0.59	0.03 ± 0.002 0.10 ± 0.007 9.34 ± 0.39	>0.99 >0.99 0.78
**Salivary analysis**: • Cortisol (ng/ml) • SAA (U/ml) • sIgA (µg/ml)	4.88 ± 0.45 102.92 ± 23.45 192.84 ± 18.10	5.81 ± 0.81 76.44 ± 12.76 164.48 ± 12.16	0.34 0.32 0.21
**Study questionnaires**: • STAI • PSS	29.80 ± 0.84 7.89 ± 0.69	28.74 ± 0.94 7.85 ± 0.72	0.41 0.92

**Abbreviations**: BMI, Body Mass Index; FEL, Fractional Excretion of Lactulose; FEM, Fractional Excretion of Mannitol; GAD-7, General Anxiety Disorder7-item; LMR, Lactulose Mannitol ratio; NA, not applicable; PHQ-9, Patient Health Questionnaire 9-item; PSS, Perceived Stress Scale; SAA, Salivary Alpha Amylase; sIgA, secretory IgA; STAI, State Trait Anxiety Inventory.

### Primary outcome (LMR)

Main and interaction effects of all outcomes are shown in Table 2. The hypothesis of a decrease in stress-related intestinal permeability quantified by the LMR with the *L. rhamnosus*-containing product vs. placebo was refuted based on the absence of stress-induced changes in LMR or difference with the test product (p = .9) vs. placebo (p = .69) (p = .71 for interaction). Results were similar when adjusted for age, gender, study topic and degree, as well as in subjects with a stress-induced cortisol above the 90th percentile (P90) of baseline (see **Supplementary material**).

**Table 2.** Main and interaction effects for outcomes in the intention-to-treat analysis

Effect	Visit		Treatment		Visit*treatment	
**Outcome**	**F value**	**p value**	**F value**	**p value**	**F value**	**p value**
**Primary**: - LMR	56.94	<0.0001	1.52	0.22	0.80	0.50
**Secondary**: - Cortisol - STAl	104.27 113.98	<0.0001 <0.0001	0.58 0.02	0.45 0.88	0 2.61	0.97 0.05
**Exploratory**: - SAA - sIgA - FEL - FEM - PSS	71.34 48.14 30.91 5.16 11.23	<0.0001 <0.0001 <0.0001 0.002 <0.0001	0.79 0.53 0.45 0.42 0.21	0.38 0.47 0.50 0.52 0.65	3.51 0.25 0.48 1.40 1.88	0.07 0.62 0.70 0.24 0.13

**Abbreviations**: FEL, Fractional Excretion of Lactulose; FEM, Fractional Excretion of Mannitol; LMR, Lactulose Mannitol ratio; PSS, Perceived Stress Scale; SAA, Salivary Alpha Amylase; sIgA, secretory IgA; STAI, State Trait Anxiety Inventory.

### Secondary outcomes (LMR, cortisol, and STAI)

**Figure 2.**
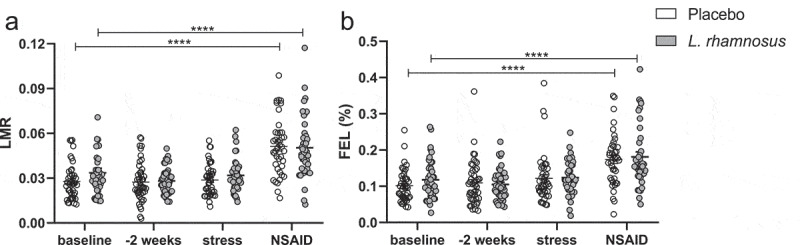
Evolution of the lactulose-mannitol ratio (a) and fractional excretion of lactulose (b) across all visits in the intention-to-treat population. LMR, lactulose-mannitol ratio; NSAID, non-steroidal anti-inflammatory drug. ****p < .0001

The pre-specified evaluation of LMR across visits showed no change after 2 weeks with the test product (p = .11) or placebo (p = .49) treatment. However, a significant but similar increase in LMR was seen after NSAID with the test product (p < .0001) vs. placebo (p < .0001) (p_adj_ = 0.28 for interaction). The evolution of LMR across visits with both treatments is illustrated in Figure 2a.

**Figure 3.**
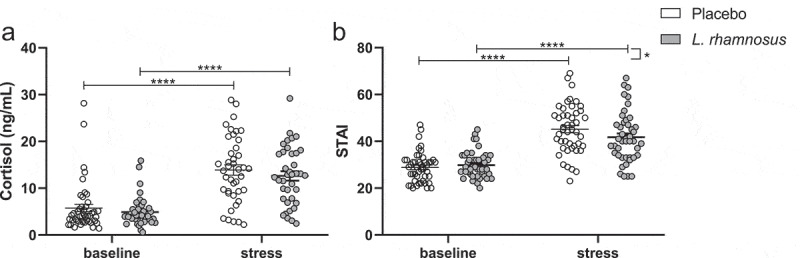
Stress-induced increase in salivary cortisol (a) and STAI (b) in the intention-to-treat population.

A significant but similar stress-induced increase in cortisol was found with the *L. rhamnosus*-containing product (p < .0001) vs. placebo (p < .0001) (p = .97 for interaction). In contrast, the stress-induced increase in STAI was significantly lower with the test product (p < .0001) vs. placebo (p < .0001) (p = .01 for interaction). The stress-induced changes in cortisol and STAI are illustrated in Figure 3.

STAI, State Trait Anxiety Inventory. *p < .05, ****p < .0001

### Exploratory outcomes (SAA, sIgA, FEL, FEM, and PSS)

The stress-induced increase in SAA tended to be lower with with the test product (p < .0001) vs. placebo (p < .0001) (p = .07 for interaction). In contrast, a similar stress-induced increase in sIgA was found with the test product (p < .0001) vs. placebo (p < .0001) (p = .62 for interaction). The stress-induced changes in sIgA and SAA are illustrated in **Figure S1** (**Supplementary material**).

**Figure 4.**
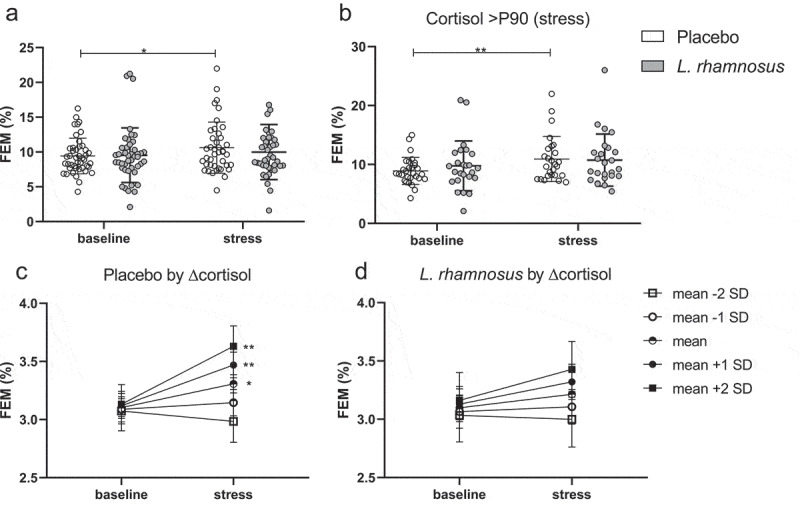
Stress-induced increase in the fractional excretion of mannitol in the intention-to-treat (a) and subgroup analysis of subjects with cortisol >P90 during stress (b). Mediation analysis with estimates at baseline and during stress for different levels of the stress-induced change in cortisol with the placebo (c) and test product (d) treatment.

**Figure 5.**
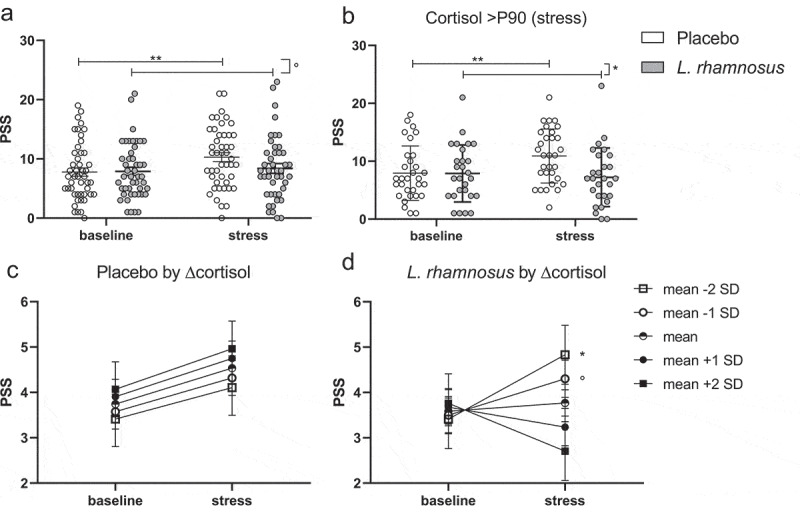
Stress-induced increase in the perceived stress scale in the intention-to-treat (a) and subgroup analysis of subjects with salivary cortisol >P90 during stress (b). Mediation analysis with estimates at baseline and stress for different levels of the stress-induced change in cortisol with the placebo (c) and test product (d) treatment.

For FEL, no changes or difference after stress but similar increases after NSAID were found with the test product (p < .0001) vs. placebo (p < .0001) (p_adj_ = 0.55 for interaction) (Figure 2b). For FEM, a stress-induced increase was seen with the placebo (p < .05) but not test product (p = .27), with no between-treatment difference (p = .59). Similarly, a stress-induced increase in PSS was found with the placebo (p = .002) but not test product (p = .73) (p = .06 for interaction). The stress-induced increase in FEM and PSS with placebo was more pronounced in the pre-specified subgroup analysis of subjects with stress-induced cortisol >P90 of baseline, with a significant between-treatment difference for PSS (see **Supplementary material**). Stress-induced increases in FEM (Figure 4a-b) and PSS (Figure 5 a-b) are illustrated for the intention-to-treat and subgroup analyses.

Graphs C-D show means and standard error of FEM after Box-Cox transformation for different levels of the standardized (mean = 0 and standard deviation = 1) stress-induced change in cortisol. P90, 90^th^ percentile; FEM, fractional excretion of mannitol. *p < .05, **p < .01

Graphs C-D show means and standard error of PSS after Box-Cox transformation for different levels of the standardized (mean = 0 and standard deviation = 1) stress-induced change in cortisol. P90, 90^th^ percentile; PSS, Perceived Stress Scale. °p < .1, *p < .05, **p < .01

### Mediation analysis

We hypothesized that the effects of the product with *L. rhamnosus* on subjective stress and mannitol excretion described above were dependent on the level of HPA-axis activation. Therefore, the mediating effect of cortisol was tested by adding the standardized stress-induced change in cortisol (Δcortisol) in the model for STAI, FEM, and PSS. No mediation was found for STAI with the placebo or test product (see **Supplementary material**). However, the stress-induced increase in FEM was only found in subjects with average or higher changes in cortisol with the placebo but not test product (Figure 4 c-d), which is in line with the subgroup analysis in subjects with stress-induced cortisol >P90 of baseline. While no mediation was found for PSS with the placebo, a stress-induced increase in PSS was only found in subjects with below average changes in cortisol with the test product, indicating a lack of preventative effect with limited HPA-axis activation (Figure 5 c-d). When adding ΔFEM in the model with STAI and PSS, no mediation was found with either the placebo or test product.

### Safety

All AE with their relation to the product are described in the **Supplementary material**.

## Discussion

In this randomized double-blind placebo-controlled trial involving healthy students, no stress-related changes in LMR were found during public speech. Hence, it was not possible to observe an effect of *L. rhamnosus* CNCM I-3690 on this parameter. Nevertheless, several important changes were found in the other pre-specified outcomes. Although both objective (cortisol) and subjective (STAI) stress significantly increased during stress, confirming the biological and behavioral stress-response to public speech, only STAI was reduced by the *L. rhamnosus* strain. Interestingly, the stress-induced increase in PSS was prevented by the test product, especially in the subgroup with a stress-induced cortisol >P90 of baseline. Moreover, the stress-induced increase in PSS was still present in subjects with below average changes in cortisol despite intake of the test product, indicating a lack of protective behavioral effects in the case of low HPA-axis activation. We also found a stress-induced increase in salivary markers of autonomic (SAA) and immune (sIgA) activation, with a trend for a smaller increase with the test product for SAA. Finally, a stress-induced increase in FEM was found with the control product only, which was mediated by cortisol levels and no mediating effect of FEM was found on subjective stress, indicating anxiolytic effects, which were independent of barrier stabilization.

Increased intestinal permeability in healthy subjects has been studied in different non-inflammatory stress conditions, including physical exercise, intake of NSAIDs and psychological stress.^19^ Although the passage of orally ingested sugars is still incompletely understood, *in vivo* assessment of intestinal barrier function allows a noninvasive measurement. It has been proposed that the monosaccharide mannitol passes the epithelium via the pore and not the leak pathway such as the disaccharide lactulose, but this concept is controversial.^19^ An alternative hypothesis is that mannitol and lactulose are markers for transcellular and paracellular passage respectively, but supporting evidence is lacking.^20^ Previous preclinical studies with *L. rhamnosus* CNCM I-3690 showed increased expression of the tight junction protein occludin, suggesting restored paracellular permeability,^16^ which was later confirmed with additional effects on mucus production by goblet cells.^17^ Interestingly, these effects were dependent on the adhesion proteins or pili structures.^17^ While similar effects on the tight junction-expression were found with *L. plantarum* strains in the small intestine of healthy volunteers, LMR was unaffected after NSAID,^21,22^ pointing to potential effects of the *L. rhamnosus* strain in humans even in the absence of changes in LMR. Notwithstanding the limitations of sugar excretion tests, alternative measures are costly and invasive and no validated blood markers for permeability are available.^23^

Besides variability in the stress-response itself, inter- and intra-individual test variability was higher for FEL and LMR compared to FEM, which is in accordance with previous studies,^20^ and may explain discrepant results on LMR with our previous study.^15^ While only a NSAID-induced increase in LMR and FEL was found in the current study, a significant stress-induced increase in FEM was found with the placebo but not test product. Our finding of increased permeability to mannitol is in agreement with previous studies in IBS patients with diarrhea and small intestinal hyperpermeability.^24^ Moreover, changes in intestinal permeability may be driven by psychological stress, as mouse models showed neuroendocrine dysfunction with mucosal barrier dysfunction after maternal deprivation as an early life stressor.^25^ Indeed, increased FEM was mediated by changes in cortisol and more pronounced in the subgroup of subjects with high cortisol levels with a trend for increased FEL with placebo treatment, suggesting an activation of the lactulose pathway only in case of more pronounced stress. Thus, it is plausible that higher levels or more protracted stress conditions are needed to activate this larger molecule pathway.

The public speech stressor used in the current study was however effective for inducing stress at a biological and behavioral level. Importantly, the stress-induced increase in STAI was significantly lower with the test product compared to the placebo treatment. Although a previous study failed to detect effects of *L. rhamnosus* JB-1 on subjective or objective stress after acute stress, the authors noted that demonstrating probiotic effects in healthy individuals is challenging.^9^ Moreover, a more artificial stressor (socially evaluated cold pressor test) was used, in contrast to a thesis defense by students in our study. Recent systematic reviews concluded that probiotics and particularly *L. rhamnosus* may improve anxiety in rodents,^5,6^ and reasons for conflicting results may lie in the stressors used and subjects’ background. In this study, increased anxiety and perceived stress levels were found with the placebo, independently of the change in cortisol, pointing toward the strong anticipatory effect of public speech on behavioral and biological level. Interestingly, the lack of stress-preventive effects of the test product on PSS in case of below average changes in cortisol suggests that this strain is less effective in the absence of HPA-axis activation. Although discrepancies between biological and behavioral stress-responses are common,^26^ we speculate that lower HPA-axis activation may be indicative of a lack of anticipatory stress to public speech.

Probiotics may affect gut-brain signaling independent of changes in permeability.^27^ Indeed, although the test product prevented a stress-induced increase in FEM, no mediating effect of FEM was found for STAI and PSS with placebo treatment, suggesting that barrier stabilization by *L. rhamnosus* CNCM I-3690 is not critical for its stress-mitigating effect. Altered central expression of gamma-aminobutyric acid (GABA)-receptors by *L. rhamnosus* JB-1 was dependent on the vagus nerve,^7^ which was also involved in the anxiolytic effects of *B. longum* NCC3001.^28^ Indeed, *Lactobacillus*-species produce multiple neurotransmitters and neuronal effects of *L. rhamnosus* were previously reported.^27,29^ Moreover, gut-brain signaling of short-chain fatty acids (SCFA), neurohumoral signaling molecules produced by different probiotic strains, may be independent of gut barrier function.^30^ The association between particular bacterial genera and quality of life or depression was also explained by decreased butyrate-production.^31^ Although we did not measure upstream mediators of (vagal) signaling, a trend for a lower stress-induced increase with probiotic vs. placebo was found for SAA, which is a marker of autonomic nervous system activation. However, as the secretory immune response decreased with a chronic stressor,^32^ these additional probiotic effects may only be found in case of chronic and not acute stress.

There are several limitations of the current study. As discussed above, we were limited to noninvasive tests of permeability in this large study with healthy students, and limitations inherent to the LMR could not be completely avoided. Despite the exclusion of medical diets, differences in diet may still exist and were not recorded. While we focused on acute stress levels, we did not measure the recovery phase of the stress-response, requiring repeated sampling post-stress. Although decreased subjective stress levels were found after 4-week intake, a longer intervention period could be necessary to detect biological effects as reduced salivary cortisol was only found after 8 weeks in a Japanese study.^33^ Moreover, replication of our results in an independent study powered for detecting differences in subjective stress is warranted.

Strengths of the current study include the homogenous study population with large sample size and the used stress paradigm. The use of a naturalistic social stressor, which was not planned for the purpose of the current study, was effective in our previous study and most likely contributes to the fact that this is the first human study indicating stress- and anxiety-reducing effects of *L. rhamnosus*, confirming its potential as a ‘psychobiotic’. As increased permeability is more likely a consequence than cause of stress, future studies should include markers of neurohumoral modulation (eg. neurotransmitters or SCFA), to identify possible underlying mechanisms and predictors of response.

In conclusion, we present the first clinical evidence of reduced subjective but not objective markers of stress with *L. rhamnosus* CNCM I-3690, which was safe and well tolerated. These anxiolytic effects were independent of barrier-protective effects and thus attractive for the reduction of subjective stress in both GI disorders and health.

## Materials and methods

### Study design and procedures

**Figure 6.**
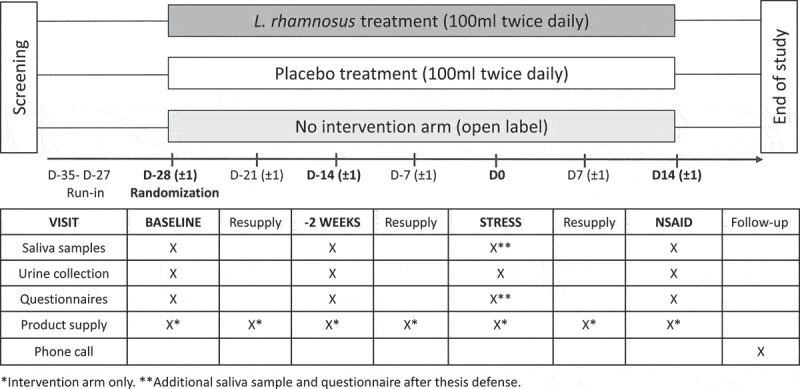
Study design.

The design of this single-center study with a randomized, controlled and parallel-group design (registered with Clinicaltrials.gov on January 17, 2018, NCT03408691) is shown in Figure 6. An active (*L. rhamnosus-*containing test product) and control (placebo product) intervention as well as a no-intervention (open label) group were included in a 2:2:1 ratio. The open label arm was added to confirm our previous data on the impact of psychological stress on intestinal permeability, and to exclude an effect of dairy product in the control or placebo treatment.^15^ All visits were scheduled according to the planned public thesis defense (D0). This was a scheduled public speech (bachelor or master thesis) in English and in front of an examination jury followed by questions with a total duration between 30 and 45 min.

NSAID, non-steroidal anti-inflammatory drug.

After a screening visit for eligibility, a run-in period of minimum 15 days took place prior to randomization. A baseline visit >1 month (D-35 to D-27) and a second visit 2 weeks before the thesis defense (D-14 ± 1 day) were planned with collection of samples and questionnaires. On the day of the thesis defense (D0), additional samples and questionnaires were obtained to evaluate the effect of the stressor. A final visit was planned 2 weeks after the thesis (D + 14 ± 1 day), with routine procedures after intake of indomethacin, a non-steroidal anti-inflammatory drug (NSAID), as a positive control to increase the intestinal permeability.^15^ This was followed by a safety follow-up phone call after 1 to 2 weeks to check for potential adverse events. The trial was conducted according to the Declaration of Helsinki and Good Clinical Practice regulations after approval by the Ethics Committee of University Hospitals Leuven (number S60969). Written informed consent was obtained from each study participant before inclusion. All data were collected at KU Leuven and University Hospitals Leuven (Leuven, Belgium).

### Study participants

Subjects were healthy female or male students, aged 20–30 years old, who were recruited from the faculty of (bio-)medical and pharmaceutical sciences or industrial engineering (bachelor or master). Structured medical history and psychiatric screening was performed to exclude chronic GI disorders or psychiatric diseases, with scores of ≥10 on the General Anxiety Disorder 7-item (GAD-7) and Patient Health Questionnaire 9-item (PHQ-9) scales leading to exclusion.^34,35^ A diagnosis of medical conditions such as atopic conditions requiring active treatment, diabetes mellitus (type 1 or 2) and/or a first degree relative with type 1 diabetes, celiac disease or IBD were exclusionary. Subjects with food allergy or hypersensitivity to any component of the products and known or suspected lactose intolerance were also excluded.

### Study products and dispensing

The test product was a non-commercialized fermented dairy product containing *L. rhamnosus* CNCM I-3690 (10^11^ CFU/100 g) (Danone culture collection, deposited in the public collection CNCM of Institut Pasteur). The control product was a non-fermented dairy product acidified with ortho-phosphoric acid, with the same packaging and matched for color, texture, taste, and nutritional content. Both the test and control product contained the following additives authorized for use in food for human consumption: milk, cream, skim milk powder or water, lactose, and fruit cocktail flavor with additionally ortho-phosphoric acid and 0.6% carboxy-methyl cellulose in the control product (see **Supplementary material**). Study products were consumed as one bottle (100 ml) twice daily. Good compliance was defined as product intake between 80% and 120%.

### Sample collection and processing

#### In vivo permeability testing

Lactulose and mannitol concentrations were determined by a HPLC-ELSD method (high-performance liquid chromatography with evaporative light scattering detector) in a 2 h-urine collection after ingestion of 5 g of lactulose and 2 g of mannitol in 200 mL of water as previously reported.^15^ LMR was determined as the concentration of lactulose divided by mannitol. The fractional excretion of lactulose (FEL) and mannitol (FEM) were also calculated.

#### Salivary markers of objective stress

Salivary cortisol was measured as a marker of the hypothalamic-pituitary-adrenal (HPA) axis-activation. Salivary Alpha-Amlyase (SAA) and secretory IgA (sIgA) were measured as markers of the autonomic and immune component of the stress response,^36,37^ respectively.

#### Psychological questionnaires

Momentary anxiety levels were measured with the state version of the validated State-Trait Anxiety Inventory (STAI) questionnaire.^38^ Perceived stress in the preceding week was assessed with the 10-item Perceived Stress Scale (PSS).^39^ Both questionnaires were collected before the urine collection on each test day with an additional STAI immediately after the thesis.

### Study outcomes

The primary endpoint was the effect of the test product containing *L. rhamnosus* CNCM I-3690 compared to the control product (placebo) on the stress-induced change in LMR from baseline. Secondary endpoints were the change of LMR after 2 weeks and NSAID vs. baseline, as well as salivary cortisol and STAI during stress vs. baseline within- and between-treatments. Pre-specified exploratory outcomes were the change of SAA, sIgA, FEL, FEM, and PSS during stress vs. baseline within- and between-treatments.

### Randomization and blinding

Randomization was performed after the baseline visit using an Interactive Web Response System of Venn Life Sciences SAS (Paris, France). Randomization was not stratified and the list was generated with a block size of 5. All study participants and on-site study personnel remained blinded for the treatment in the intervention arms (test or control product).

### Sample size

Based on the results of our previous study with DSCG,^15^ we hypothesized a 50% reduction of the stress-induced increase in LMR in the test compared to control product (placebo). Using a randomization ratio of 1:1 for test and placebo product with a type 1 error of 0.05, a total number of 72 evaluable subjects (36;36) allowed a power of 0.88. During the study, the target recruitment number was increased to 96 subjects (48;48) to allow a power of 0.95 for a 50% reduction and a power of 83% for 40% reduction of the stress-related permeability defect.

### Safety

Subjects were questioned about possible adverse events (AE) at every study visit and during a safety follow-up phone call 1 to 2 weeks after the final visit (earlier if premature termination of the study). AE were graded using the Common Terminology Criteria for Adverse Events version 4.0 and reported for all randomized subjects.

### Statistical analysis

Data from the full analysis set, including subjects randomized and exposed to products in the intervention arms, were analyzed according to the intention-to-treat principle. Baseline variables were compared with the Mann–Whitney *U* test for continuous data and chi-square test for proportions. Outcome or dependent variables were analyzed using linear mixed models, with test visits (baseline, −2 weeks, stress and NSAIDs) as within- and treatment (test product or placebo) as between-subject independent variables of interest with their interaction, indicating between-treatment differences. The assumption of a normal distribution (based on the Kolmogorov–Smirnov test) was checked for all outcomes, with Box-Cox or logarithmic transformations of the outcome variable to normalize this distribution. The variance-covariance structure providing the best fit was chosen based on the lowest value of the Akaike’s information criterion. All data were analyzed in SAS 9.4 (SAS Institute, Cary, USA) and least-squares means estimates are given as mean ± standard error (SE). A two-tailed p-value <0.05 was considered significant and 0.05 < p < .10 a trend. Results are reported in accordance with 2010 CONSORT guidelines.^40^ Additional methodological details can be found in the **Supplementary material**.

## Supplementary Material

Supplemental Material

## Data Availability

The datasets generated and analyzed during the current study are available upon reasonable request to the corresponding author and after a data transfer agreement has been signed, in line with the ethical protocol. https://clinicaltrials.gov/ct2/show/NCT03408691.

## Abbreviations

AE, adverse event; BMI, Body Mass Index; CRH, corticotropin-releasing hormone; FEL, Fractional Excretion of Lactulose; FEM, Fractional Excretion of Mannitol; GI, gastrointestinal; HPA, hypothalamic-pituitary-adrenal; IBD, inflammatory bowel disease; IBS, irritable bowel syndrome; LMR, Lactulose mannitol ratio; NSAID, Non-Steroidal Anti-Inflammatory Drug; PSS, Perceived Stress Scale; SAA, Salivary Alpha Amylase; SE, standard error; sIgA, Secretory IgA; STAI, State Trait Anxiety Inventory.

## References

[1] Collins SM, Surette M, Bercik P. The interplay between the intestinal microbiota and the brain. Nat Rev Microbiol. 2012;10(11):735–12. doi:10.1038/nrmicro2876. 23000955

[2] Cryan JF, Dinan TG. Mind-altering microorganisms: the impact of the gut microbiota on brain and behaviour. Nat Rev Neurosci. 2012;13(10):701–712. doi:10.1038/nrn3346. 22968153

[3] Sudo N, Chida Y, Aiba Y, Sonoda J, Oyama N, Yu X-N, Kubo C, Koga Y. Postnatal microbial colonization programs the hypothalamic-pituitary-adrenal system for stress response in mice. J Physiol. 2004;558(1):263–275. doi:10.1113/jphysiol.2004.063388. 15133062 PMC1664925

[4] Hill C, Guarner F, Reid G, Gibson GR, Merenstein DJ, Pot B, Morelli L, Canani RB, Flint HJ, Salminen S, et al. Expert consensus document: the international scientific association for probiotics and prebiotics consensus statement on the scope and appropriate use of the term probiotic. Nat Rev Gastroenterol Hepatol. 2014;11(8):506–514. doi:10.1038/nrgastro.2014.66. 24912386

[5] Reis DJ, Ilardi SS, Punt SEW. The anxiolytic effect of probiotics: a systematic review and meta-analysis of the clinical and preclinical literature. PLoS ONE. 2018;13(6):e0199041. doi:10.1371/journal.pone.0199041. 29924822 PMC6010276

[6] Vitellio P, Chira A, De Angelis M, Dumitrascu DL, Portincasa P. Probiotics in psychosocial stress and anxiety. A Syst Rev J Gastrointestin Liver Dis. 2020;29(1):77–83. doi:10.15403/jgld-352. 32176751

[7] Bravo JA, Forsythe P, Chew MV, Escaravage E, Savignac HM, Dinan TG, Bienenstock J, Cryan JF. Ingestion of lactobacillus strain regulates emotional behavior and central gaba receptor expression in a mouse via the vagus nerve. Proc Natl Acad Sci. 2011;108(38):16050–16055. doi:10.1073/pnas.1102999108. 21876150 PMC3179073

[8] Neufeld KA M, Kay S, Bienenstock J. Mouse strain affects behavioral and neuroendocrine stress responses following administration of probiotic lactobacillus rhamnosus JB-1 or traditional antidepressant fluoxetine. Front Neurosci. 2018;12:294. doi:10.3389/fnins.2018.00294. 29867313 PMC5952003

[9] Kelly JR, Allen AP, Temko A, Hutch W, Kennedy PJ, Farid N, Murphy E, Boylan G, Bienenstock J, Cryan JF, et al. Lost in translation? the potential psychobiotic lactobacillus rhamnosus (jb-1) fails to modulate stress or cognitive performance in healthy male subjects. Brain Behav Immun. 2017;61:50–59. doi:10.1016/j.bbi.2016.11.018. 27865949

[10] Söderholm JD, Yang P-C, Ceponis P, Vohra A, Riddell R, Sherman PM, Perdue MH. Chronic stress induces mast cell-dependent bacterial adherence and initiates mucosal inflammation in rat intestine. Gastroenterol. 2002;123(4):1099–1108. doi:10.1053/gast.2002.36019. 12360472

[11] Agostini S, Goubern M, Tondereau V, Salvador-Cartier C, Bezirard V, Lévèque M, Keränen H, Theodorou V, Bourdu-Naturel S, Goupil-Feuillerat N, et al. A marketed fermented dairy product containing bifidobacterium lactis cncm i-2494 suppresses gut hypersensitivity and colonic barrier disruption induced by acute stress in rats. Neurogastroenterol Motil. 2012;24(4):376–e172. doi:10.1111/j.1365-2982.2011.01865.x. 22272920

[12] Ait-Belgnaoui A, Durand H, Cartier C, Chaumaz G, Eutamene H, Ferrier L, Houdeau E, Fioramonti J, Bueno L, Theodorou V. Prevention of gut leakiness by a probiotic treatment leads to attenuated hpa response to an acute psychological stress in rats. Psychoneuroendocrinol. 2012;37(11):1885–1895. doi:10.1016/j.psyneuen.2012.03.024. 22541937

[13] Wallon C, Persborn M, Jönsson M, Wang A, Phan V, Lampinen M, Vicario M, Santos J, Sherman PM, Carlson M, et al. Eosinophils Express Muscarinic Receptors and Corticotropin-Releasing Factor to Disrupt the Mucosal Barrier in Ulcerative Colitis. Gastroenterol. 2011;140(5):1597–1607. doi:10.1053/j.gastro.2011.01.042. 21277851

[14] Bednarska O, Walter SA, Casado-Bedmar M, Ström M, Salvo-Romero E, Vicario M, Mayer EA, Keita ÅV. vasoactive intestinal polypeptide and mast cells regulate increased passage of colonic bacteria in patients with irritable bowel syndrome. Gastroenterol. 2017;153(4):948–960.e3. doi:10.1053/j.gastro.2017.06.051. PMC5623149 28711627

[15] Vanuytsel T, van Wanrooy S, Vanheel H, Vanormelingen C, Verschueren S, Houben E, Salim Rasoel S, Tόth J, Holvoet L, Farré R, et al. psychological stress and corticotropin-releasing hormone increase intestinal permeability in humans by a mast cell-dependent mechanism. Gut. 2014;63(8):1293–1299. doi:10.1136/gutjnl-2013-305690. 24153250

[16] Laval L, Martin R, Natividad JN, F Chain FC, Miquel S, Desclée de Maredsous C, Capronnier S, Sokol H, Verdu EF, van Hylckama Vlieg JET, et al. lactobacillus rhamnosus cncm i-3690 and the commensal bacterium faecalibacterium prausnitzii a2–165 exhibit similar protective effects to induced barrier hyper-permeability in mice. Gut Microbes. 2015;6(1):1–9. doi:10.4161/19490976.2014.990784. 25517879 PMC4615674

[17] Martín R, Chamignon C, Mhedbi-Hajri N, Chain F, Derrien M, Escribano-Vázquez U, Garault P, Cotillard A, Pham HP, Chervaux C, et al. The potential probiotic lactobacillus rhamnosus CNCM I-3690 strain protects the intestinal barrier by stimulating both mucus production and cytoprotective response. Sci Rep. 2019;9(1):5398. doi:10.1038/s41598-019-41738-5. 30931953 PMC6443702

[18] Natividad JM, Lamas B, Pham HP, Michel ML, Rainteau D, Bridonneau C, Da Costa G, Van Hylckama Vlieg J, Sovran B, Chamignon C, et al. Bilophila wadsworthia aggravates high fat diet induced metabolic dysfunctions in mice. Nat Commun. 2018;9(1):2802. doi:10.1038/s41467-018-05249-7. 30022049 PMC6052103

[19] Camilleri M. Leaky gut: mechanisms, measurement and clinical implications in humans. Gut. 2019;68(8):1516–1526. doi:10.1136/gutjnl-2019-318427. 31076401 PMC6790068

[20] Blomquist L, Bark T, Hedenborg G, Svenberg T, Norman A. Comparison between the lactulose/mannitol and 51Cr-ethylenediaminetetraacetic acid/14C-mannitol methods for intestinal permeability. frequency distribution pattern and variability of markers and marker ratios in healthy subjects. Scand J Gastroenterol. 1993;28(3):274–280. doi:10.3109/00365529309096085. 8446853

[21] Karczewski J, Troost FJ, Konings I, Dekker J, Kleerebezem M, Brummer RJM, Wells JM. Regulation of human epithelial tight junction proteins by lactobacillus plantarum in vivo and protective effects on the epithelial barrier. Am J Physiol Gastrointest Liver Physiol. 2010;298(6):G851–G859. doi:10.1152/ajpgi.00327.2009. 20224007

[22] Mujagic Z, De Vos P, Boekschoten MV, Govers C, Pieters HJHM, De Wit NJW, Bron PA, Masclee AAM, Troost FJ. The effects of lactobacillus plantarum on small intestinal barrier function and mucosal gene transcription; a randomized double-blind placebo controlled trial. Sci Rep. 2017;7(1):40128. doi:10.1038/srep40128. 28045137 PMC5206730

[23] Vanuytsel T, Tack J, Farré R. the role of intestinal permeability in gastrointestinal disorders and current methods of evaluation. Front Nutr. 2021;8::717925;. doi:10.3389/fnut.2021.717925. PMC8427160 34513903

[24] Rao AS, Camilleri M, Eckert DJ, Busciglio I, Burton DD, Ryks M, Wong BS, Lamsam J, Singh R, Zinsmeister AR. Urine sugars for in vivo gut permeability: validation and comparisons in irritable bowel syndrome-diarrhea and controls. Am J Physiol Gastrointest Liver Physiol. 2011;301(5):G919–28. doi:10.1152/ajpgi.00168.2011. 21836056 PMC3220318

[25] Barreau F, Cartier C, Leveque M, Ferrier L, Moriez R, Laroute V, Rosztoczy A, Fioramonti J, Bueno L. Pathways involved in gut mucosal barrier dysfunction induced in adult rats by maternal deprivation: corticotrophin-releasing factor and nerve growth factor interplay. J Physiol. 2007;580(1):347–356. doi:10.1113/jphysiol.2006.120907. 17234701 PMC2075424

[26] Campbell J, Ehlert U. Acute psychosocial stress: does the emotional stress response correspond with physiological responses? Psychoneuroendocrinol. 2012;37(8):1111–1134. doi:10.1016/j.psyneuen.2011.12.010. 22260938

[27] Kelly JR, Kennedy PJ, Cryan JF, Dinan TG, Clarke G, Hyland NP. Breaking down the barriers: the gut microbiome, intestinal permeability and stress-related psychiatric disorders. Front Cell Neurosci. 2015;9:392. doi:10.3389/fncel.2015.00392. 26528128 PMC4604320

[28] Bercik P, Park AJ, Sinclair D, Khoshdel A, Lu J, Huang X, Deng Y, Blennerhassett PA, Fahnestock M, Moine D, et al. The anxiolytic effect of *bifidobacterium longum* NCC3001 involves vagal pathways for gut-brain communication. Neurogastroenterol Motil. 2011;23(12):1132–1139. doi:10.1111/j.1365-2982.2011.01796.x. 21988661 PMC3413724

[29] Perez-Burgos A, Mao YK, Bienenstock J, Kunze WA. The gut-brain axis rewired: adding a functional vagal nicotinic “sensory synapse. FASEB J. 2014;28(7):3064–3074. doi:10.1096/FJ.13-245282. 24719355

[30] Dalile B, Van Oudenhove L, Vervliet B, Verbeke K. The role of short-chain fatty acids in microbiota–gut–brain communication. Nat Rev Gastroenterol Hepatol. 2019;16(8):461–478. doi:10.1038/s41575-019-0157-3. 31123355

[31] Valles-Colomer M, Falony G, Darzi Y, Tigchelaar EF, Wang J, Tito RY, Schiweck C, Kurilshikov A, Joossens M, Wijmenga C, et al. the neuroactive potential of the human gut microbiota in quality of life and depression. Nat Microbiol. 2019;4(4):623–632. doi:10.1038/s41564-018-0337-x. 30718848

[32] Engeland CG, Hugo FN, Hilgert JB, Nascimento GG, Junges R, Lim H-J, Marucha PT, Bosch JA. psychological distress and salivary secretory immunity. Brain Behav Immun. 2016;52:11–17. doi:10.1016/j.bbi.2015.08.017. 26318411 PMC5841158

[33] Kato-Kataoka A, Nishida K, Takada M, Kawai M, Kikuchi-Hayakawa H, Suda K, Ishikawa H, Gondo Y, Shimizu K, Matsuki T, et al. Fermented milk containing lactobacillus casei strain shirota preserves the diversity of the gut microbiota and relieves abdominal dysfunction in healthy medical students exposed to academic stress. Appl Environ Microbiol. 2016;82(12):3649–3658. doi:10.1128/AEM.04134-15. 27208120 PMC4959178

[34] Kroenke K, Spitzer RL, Williams JBW. The PHQ-9: validity of a brief depression severity measure. J Gen Intern Med. 2001;16(9):606–613. doi:10.1046/j.1525-1497.2001.016009606.x. 11556941 PMC1495268

[35] Spitzer RL, Kroenke K, Williams JBW, Löwe B. A Brief Measure for Assessing Generalized Anxiety Disorder: the GAD-7. Arch Intern Med. 2006;166(10):1092–1097. doi:10.1001/archinte.166.10.1092. 16717171

[36] Granger DA, Kivlighan KT, El-Sheikh M, Gordis EB, Stroud LR. Salivy α-Amylase in Biobehavioral Research: recent Developments and Applications. Ann N Y Acad Sci. 2007;1098(1):122–144. doi:10.1196/annals.1384.008. 17332070

[37] Laurent HK, Stroud LR, Brush B, D’Angelo C, Granger DA. Secretory IgA reactivity to social threat in youth: relations with HPA, ANS, and behavior. Psychoneuroendocrinol. 2015;59:81–90. doi:10.1016/j.psyneuen.2015.04.021. PMC4490024 26036453

[38] Spielberger C, Gorsuch R, and Lushene R. Manual for the state-trait anxiety inventory (form Y self-evaluation questionnaire). Sunnyvale (CA): In Consulting Psychologists Press; 1983.

[39] Taylor JM. Psychometric analysis of the ten-item perceived stress scale. Psychol Assess. 2015;27(1):90–101. doi:10.1037/a0038100. 25346996

[40] Schulz KF, Altman DG, Moher DCONSORT. Statement: updated guidelines for reporting parallel group randomised trials. BMJ. 2010;340(7748):698–702. doi:10.1136/bmj.c332. PMC3043330 21350618

