# Supplementary material for: Lactobacillus rhamnosus CNCM I-3690 decreases subjective academic stress in healthy adults: a randomized placebo-controlled trial
Source: Gut Microbes. 2022 Feb 7;14(1):2031695. doi: 10.1080/19490976.2022.2031695 (PMC8824214; doi:10.1080/19490976.2022.2031695)
Supplement: Supplemental Material [file KGMI_A_2031695_SM3803.docx]

**Supplementary results**

***Participants and adherence***

Reasons for drop-out with the test product treatment were an unrelated adverse event and withdrawal by the subject with intake of forbidden medication (both n= 1). One subject randomized to the placebo treatment did not show up for product dispensing and was not exposed to the study product, leading to 46 subjects in the test product and placebo treatment for the intention-to-treat analysis. Proportions of study degree (master 54% vs. 45%, p= 0.53) or topic (Biomedical Sciences 50% vs. 65%, p= 0.14) and compliance (99.59 ± 0.40 % vs. 99.61 ± 0.29 %, p> 0.99) were similar in the test vs. control product group.

***Secondary and subgroup analyses (all outcomes)***

Secondary analyses of the primary outcome, adjusted for age, gender, study topic and degree showed similar results (**Table S1**) with a similar stress-induced change in LMR with the test product (p= 0.91) vs. placebo (p= 0.65) (p= 0.69 for interaction). Results of main and interaction effects were also similar for all secondary and exploratory outcomes.

The cut-off for P90 of cortisol at baseline for all subjects (placebo and test product treatment) was 9.05 ng/mL, resulting in 58/92 subjects (63%) with cortisol after stress >P90 of baseline, of which 31/46 (67%) with the placebo and 27/46 (59%) test product treatment (p> 0.05, chi-square test). Subgroup analysis of the primary outcome showed similar results compared to the first model (**Table S2**) with no stress-induced changes in LMR or difference with the test product (p= 0.97) vs. placebo (p= 0.75) (p= 0.80 for interaction). For STAI, stress-induced changes were also lower with test product vs. placebo (p= 0.05 for interaction) in subjects with cortisol >P90 during stress.

Interestingly, a trend for a stress-induced increase in FEL was found with the placebo (p= 0.08) and not test product (p= 0.68) (p= 0.38 for interaction) in subjects with stress-induced cortisol >P90 of baseline. The stress-induced increase in FEM was more pronounced with the placebo (p= 0.005) and not test product (p= 0.16) (p= 0.55 for interaction) in the subgroup analysis. Similarly, the stress-induced increase in PSS was also more pronounced with the placebo (p= 0.002) but not test product (p= 0.47), with a significant between-treatment difference (p= 0.01) in the subgroup of subjects with cortisol >P90.

***Mediation analysis***

Results for the model with STAI, FEM and PSS including the standardized Δcortisol per treatment are shown in **Table S3**. For STAI, a significant effect of visit but not Δcortisol or interaction effect (Δcortisol*visit) was found with the placebo or test product treatment, excluding mediation. For FEM, the visit and interaction effect with placebo was explained by a stress-induced increase in FEM for Δcortisol=0 (p= 0.02), +1 (p= 0.003) and +2 (p= 0.008). No main or interaction effects were found for the test product treatment. For PSS, a significant effect of visit but not Δcortisol or interaction effect was found with the placebo treatment. In contrast, the significant interaction effect with the test product was explained by a stress-induced increase in PSS for Δcortisol=-2 (p= 0.03) and -1 (p= 0.06) only. Finally, a significant visit but not ΔFEM or interaction effect (ΔFEM*visit) was found for STAI with the placebo (F= 113.1, p< 0.0001) and test product (F= 63.3, p< 0.0001) treatment. For PSS, a significant visit effect was found only for the placebo (F= 17.7, p= 0.0002) and not test product treatment with no ΔFEM or interaction effects, indicating no mediation.

***No intervention arm***

Mixed models for the open label arm with visit as independent variable of interest confirmed a significant increase in LMR after NSAID (p< .0001) but not after the thesis defense (p= .86) compared to baseline. In addition, a stress-induced increase from baseline was confirmed for salivary cortisol (p< .0001), STAI (p< .0001), SAA (p= .02) and sIgA (p= .0005) with a trend for FEM (p= .07) but not FEL (p= .11) or PSS (p= .17).

***Safety***

All AE with their relation to the product are given in **Table S4**. No AE was scored as serious and severity was always mild. None of the AE were related to the study procedures. Concerning AE with the test and placebo product treatment, a total of 43 (30 and 13 respectively) were unlikely and 17 (4 and 13 respectively) possibly related to the product. GI side effects occurred in both treatment groups. No significant differences in individually reported AE were found between treatment groups, except for a significantly higher proportion of subjects with headache in the *L. rhamnosus*-containing product vs. placebo (p=0.003, chi-square test), which was assessed as unlikely related to the test product.

**Supplementary methods**

***Study design and procedures***

Urine pregnancy tests were performed in females during screening and repeated once every month. Medication use was re-assessed at every study visit and reported using the WHO ATC/DDD Index.^1^ Dietary instructions during the study included no fermented dairy products (yoghurts, fresh cheese etc.) or probiotic supplements during the run-in and evaluation periods. Subjects on medical diets or with recent changes or plans for modifications in dietary habits were also not eligible. Intake of antibiotics, antiseptics or anti-inflammatory drugs <2 weeks before the study were not eligible and intake of these and/or relaxing, calming or stimulating drugs were not allowed during the study. Smoking and alcohol were allowed if <7 cigarettes per week and no regular intake of >3 units per day, respectively. Consumption of coffee and tea was also minimized during run-in and evaluation periods. Guidelines applied to all subjects, including the intervention (test and control product) and no-intervention (open label) groups.

Indomethacin was used as positive control. Subjects were asked to take 3 capsules of 25mg of indomethacin with 1 glass of water on the evening before the final test day (10 PM) and 2 capsules of 25mg on the morning of the test day (10 AM). This dose (125mg) is within the recommended dose range (50-200mg per day) with only mild possible adverse events including abdominal symptoms such as nausea, abdominal discomfort, diarrhea and pain or allergic reactions. Indomethacin was produced in capsules of 25mg by the pharmacy of the Leiden Universitair Medisch Centrum (LUMC, Leiden, The Netherlands) in packages of 5 capsules.

***Study products and dispensing***

*L. rhamnosus* is a bacterial species with a Generally Recognized as Safe (GRAS) status from the US Food and Drug Administration (FDA) and Qualify Presumption of Safety (QPS) status from the European Food Safety Agency (EFSA). The composition of the *L. rhamnosus* (test) and placebo product are shown in **Table S5**. All products were manufactured in a pilot plant at Danone Nutricia Research Centre (Palaiseau, France), approved by the authorities for producing dairy products for human consumption (F 91 477 011 CE) in accordance with standard procedures and food safety requirements.

Shipment to the study site was done on a weekly basis in blinded packaging with temperature logging. Subjects in the intervention arm received the allocated products in carton boxes of 20 plastic alimentary bottles containing 100ml of product, sealed with an aluminum lid. Cooling bags were provided for cooled transport with a maximum travel time of 4 hours, before storage at 2-8°C with a shelf life of 35 days. All subjects started the consumption of the study product 4 weeks before the planned thesis defense (stress), by product dispensing after randomization in the intervention arm, which was repeated weekly between (D-21, D-7 and D+7) and on each study visit (+/-1day) (**Figure 6**). Subjects were provided cooling bags with a new carton box of the study product, of which all unconsumed bottles were returned and counted on a weekly basis at the study site.

***Sample collection and processing***

*In vivo permeability testing*

A light lactose-free breakfast and intake of the study product was allowed until 6 hours before the planned test visit. Subjects were allowed to drink water until 30 min before the test visit and 250mL of water after ingestion of the sugar solution. No other food or drinking was allowed during the urine collection during each test visit. All test visits were planned in the afternoon, including the planned thesis defense. Chewing gum was not permitted on the day before and the day of a test visit, to avoid mannitol contamination. The pre-weighed plastic container contained 750mg of neomycin to prevent bacterial degradation of lactulose and mannitol and after calculating the 2h-urine volume, samples were filtered with 450 nm filters (Merck Millipore, Billerica, USA) and stored at −20°C until further analysis. Urinary lactulose and mannitol concentrations were measured using blinded sample codes with HPLC-ELSD equipped with a Shodex Asahipak column (250mmx4.6mm, 5µm particle size; BGB Analytik Benelux, Harderwijk, The Netherlands) with an internal standard (cellobiose), standard curve and reference samples with known concentrations according to standardized operation procedures.^2^ Limits of detection were 1 mg/L for lactulose and 1.2 mg/L for mannitol.^2^

*Salivary markers of objective stress*

Salivary cortisol was determined before and after the urine collection on each test day, with an additional sample after the thesis defense. Cortisol samples were collected using Salivabio oral swabs (Salimetrics, LCC, Carslab, USA) and stored at −20°C after processing until analysis with enzyme-linked immunosorbent assay (ELISA) (DRG diagnostics, Marburg, Germany). Samples for SAA and sIgA were also collected before the urine collection on each test day, with an additional sample after the thesis defense. Samples were collected using salivettes and stored at −20°C after processing until analysis with ELISA (Salimetrics, LCC, Carslab, USA) according to the manufacturer’s instructions. Salivary markers after the thesis defense and STAI scores before the thesis defense were used as these were highest in our previous study.^3^

***Randomization and blinding***

The Interactive Web Response System also generated the corresponding product number for each subject in the intervention arms, by matching individual subjects with the actual product list, provided to an independent statistician by the clinical product supply supervisor at Danone Nutricia Research (Palaiseau, France). During the study, all subjects in the intervention arms and study personnel remained blinded, except for the clinical product supply supervisor who was not involved in any other aspect of the study. Allocated products were dispensed by study personnel on site on a weekly basis. Unblinding was performed at the end of the study, after database lock.

***Statistical analysis***

Changes in outcome variables were assessed between pre-specified visits and compared between treatments as detailed in the “study outcomes” section. No gatekeeping strategies were applied and all comparisons were performed at an alpha level of 5% with the exception of changes in multiple visits for each secondary or exploratory outcome, which were corrected for multiplicity using the stepdown-Holm method. No other adjustments for multiplicity were made. Pre-specified secondary analyses were performed with the change in each outcome adjusted for age, gender, study topic and degree as covariates. Similar to our previous study,^3^ a pre-specified subgroup analysis was performed to assess stress-induced changes in outcomes in subjects with a cortisol level above the 90^th^ percentile (P90) of baseline cortisol values in all subjects. This value was used as an upper limit of normal or cut-off value to divide subjects with placebo and test product treatments based on their cortisol levels after the thesis defense. A subgroup analysis was performed in subjects with cortisol values after the thesis defense >P90 from baseline. Exploratory analyses were done in case the significant main or interactions effects were not explained by pre-specified analyses, with correction for multiple testing.

In addition, based on findings of our previous study,^3^ the potential mediating effect of cortisol in the stress-induced increase in STAI, FEM and PSS was tested by entering the stress-induced change in cortisol (Δcortisol) in the model of each individual outcome per treatment (test or control product). To visualize the effect of change in cortisol, Δcortisol was standardized with mean value of 0 and standard deviation of 1, where Δcortisol= 0 corresponds to an average change in cortisol; Δcortisol= -1 or -2 to a change below average and Δcortisol= 1 or 2 to a change above average in subjects with the placebo or test product. Changes within-treatment were assessed and plotted for the different levels of Δcortisol, including the average (0) and average ± 1 or 2 standard deviations.^4^ Finally, the stress-induced change in FEM (ΔFEM) was added in the model of STAI and PSS per treatment.

**References:**

[1] WHO Collaborating Centre for Drug Statistics Methodology, Guidelines for ATC classification and DDD assignment. https://www.whocc.no/atc_ddd_index/.

[2] Houben E, Vanuytsel T, Farre R, Tack J, Verbeke K. Validation of a GC-MS and HPLC-ELSD Method to Study Intestinal Permeability. Trends Chromatogr. 2013; 8:83–96.

[3] Vanuytsel T, van Wanrooy S, Vanheel H, Vanormelingen C, Verschueren S, Houben E, Salim Rasoel S, Tόth J, Holvoet L, Farré R, et al. Psychological Stress and Corticotropin-Releasing Hormone Increase Intestinal Permeability in Humans by a Mast Cell-Dependent Mechanism. Gut 2014; 63(8):1293–1299; https://doi.org/10.1136/gutjnl-2013-305690.

[4] Van Oudenhove L, Törnblom H, Störsrud S, Tack J, Simrén M. Depression and Somatization Are Associated with Increased Postprandial Symptoms in Patients with Irritable Bowel Syndrome. Gastroenterology 2016; 150(4):866–874; https://doi.org/10.1053/j.gastro.2015.11.010.

**Figures**

*
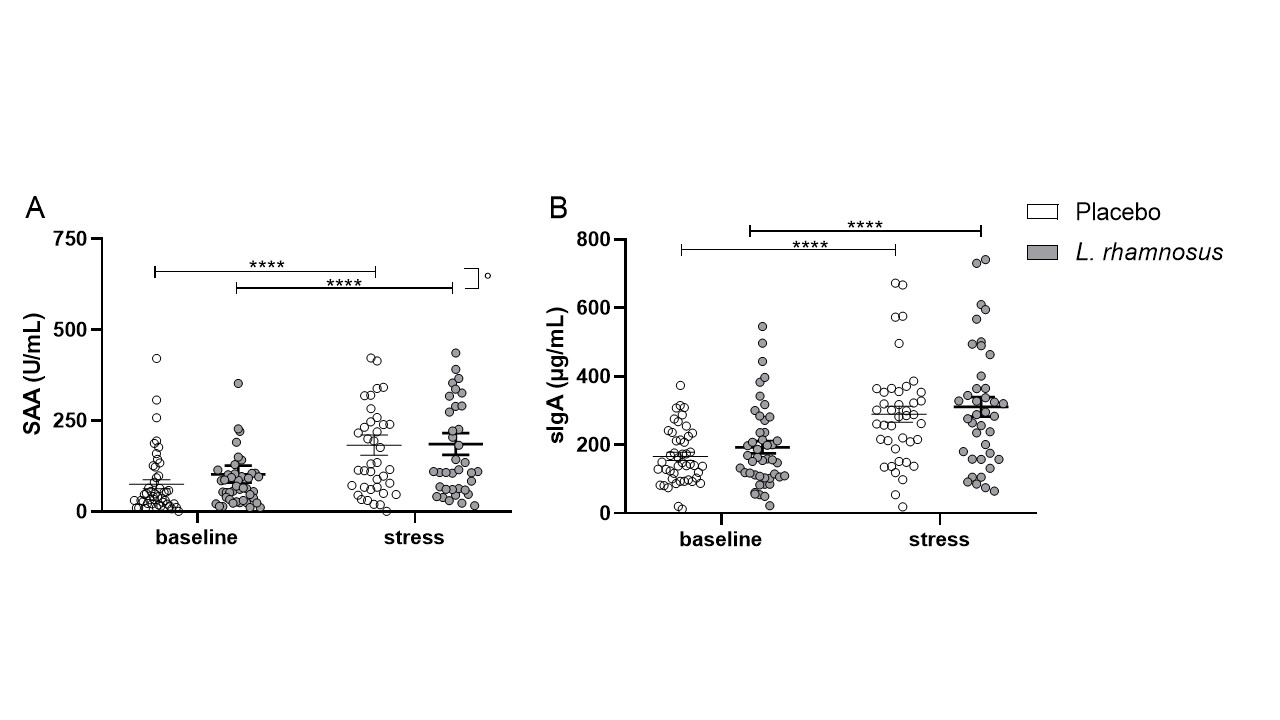
*

***Figure S1: Stress-induced increase in salivary SAA (A) and sIgA (B) in the intention-to-treat population.*** °p< 0.01, ****p< 0.0001

**Tables**

***Table S1: Main and interaction effects for secondary analyses of individual outcomes, adjusted for age, gender, study topic and degree in the intention-to-treat analysis.***

| **F value (p)** | **Visit** | **Treatment** | **Visit*treatment** | **age** | **gender** | **study topic** | **study degree** |
| --- | --- | --- | --- | --- | --- | --- | --- |
| **LMR** | 56.86 (<0.0001) | 0.43 (0.52) | 0.81 (0.49) | 4.46 (0.04) | 0.54 (0.46) | 5.77 (0.02) | 1.59 (0.21) |
| **Cortisol** | 100.85 (<0.0001) | 0.46 (0.50) | 0.02 (0.89) | 0 (0.99) | 0.01 (0.92) | 3.28 (0.07) | 1.33 (0.25) |
| **STAl** | 113.96 (<0.0001) | 0.16 (0.69) | 2.63 (0.05) | 0.3 (0.59) | 1.6 (0.21) | 5.4 (0.02) | 1.9 (0.17) |
| **SAA** | 71.08 (<0.0001) | 0.80 (0.37) | 3.49 (0.07) | 0.64 (0.43) | 0.02 (0.89) | 0.03 (0.87) | 0.90 (0.35) |
| **sIgA** | 31.04 (<0.0001) | 0.09 (0.77) | 0.50 (0.68) | 0.83 (0.36) | 3.06 (0.08) | 7.28 (0.008) | 7.33 (0.008) |
| **FEL** | 30.88 (<0.0001) | 0 (0.99) | 0.50 (0.68) | 4.62 (0.03) | 6.23 (0.01) | 5.35 (0.02) | 0.83 (0.37) |
| **FEM** | 5.18 (0.002) | 0.66 (0.42) | 1.35 (0.26) | 0.01 (0.92) | 9.69 (0.003) | 0.02 (0.90) | 0.51 (0.48) |
| **PSS** | 11.23 (<0.0001) | 0.03 (0.86) | 1.88 (0.13) | 0.34 (0.56) | 2.38 (0.13) | 4.30 (0.04) | 0.31 (0.58) |

**Abbreviations:** FEL, Fractional Excretion of Lactulose; FEM, Fractional Excretion of Mannitol; LMR, Lactulose Mannitol ratio; PSS, Perceived Stress Scale; SAA, Salivary Alpha Amylase; sIgA, secretory IgA; STAI, State Trait Anxiety Inventory.

***Table S2: Main and interaction effects for subgroup analyses of the stress-induced changes in outcomes in subjects with stress-induced cortisol >P90 of baseline.***

| **Effect** | **Visit** | | **Treatment** | | **Visit*treatment** | |
| --- | --- | --- | --- | --- | --- | --- |
| **Outcome** | **F value** | **p** | **F value** | **p** | **F value** | **p** |
| **Primary:**  - LMR | 34.43 | <0.0001 | 0.79 | 0.38 | 0.42 | 0.74 |
| **Secondary:**  - STAl | 89.62 | <0.0001 | 0.16 | 0.69 | 2.77 | 0.04 |
| **Exploratory:**  - FEL  - FEM  - PSS | 19.36  6.58  9.14 | <0.0001  0.0003  <0.0001 | 0.79  0  1.42 | 0.38  0.98  0.24 | 0.45  0.83  0.12 | 0.72  0.48  0.03 |

**Abbreviations:** FEL, Fractional Excretion of Lactulose; FEM, Fractional Excretion of Mannitol; LMR, Lactulose Mannitol ratio; PSS, Perceived Stress Scale; STAI, State Trait Anxiety Inventory.

***Table S3: Main and interaction effects after adding the standardized Δcortisol in the model per treatment (test or control product).***

| **Outcome with Δcortisol per treatment** | **F value (p)**  **visit** | **F value (p) Δcortisol** | **F value (p) Δcortisol*visit** |
| --- | --- | --- | --- |
| **Placebo:**  - STAI  - FEM  - PSS | 112.4 (<0.0001)  6.63 (0.02)  7.78 (0.008) | 2.29 (0.14)  1.76 (0.19)  0.67 (0.42) | 1.06 (0.31)  3.38 (0.08)  0.03 (0.86) |
| **Test product:**  - STAI  - FEM  - PSS | 87.2 (<0.0001)  1.06 (0.31)  0.41 (0.53) | 0.07 (0.8)  0.62 (0.44)  0.77 (0.39) | 0 (0.96)  0.42 (0.52)  4.69 (0.04) |

**Abbreviations:** FEM, Fractional Excretion of Mannitol; PSS, Perceived Stress Scale; STAI, State Trait Anxiety Inventory.

***Table S4: Adverse events per system organ class.***

|  | ***L. rhamnosus* product (n= 46)** | | **Placebo product (n= 46)** | | **Total (n= 92)** | |
| --- | --- | --- | --- | --- | --- | --- |
|  | **Event (1)** | **N (%) (2)** | **Event (1)** | **N (%) (2)** | **Event (1)** | **N (%) (2)** |
| **All** | 34 | 23 (50) | 26 | 17 (36) | 60 | 40 (43) |
| **Gastrointestinal**:  - abdominal pain  - alternating stool  - dyspepsia/ructus  - nausea  - flatulence  - gastritis(enteritis)  - stomach pain | /  /  1(**)  1(**)  /  2(**)  / | /  /  1 (2)  1 (2)  /  2 (4)  / | 1(**)  2(**)  1(**)  6(**)  1(**)  /  2(**) | 1 (2)  2 (4)  1 (2)  4 (8.5)  1 (2)  /  2 (4) | 1  2  2  7  1  2  2 | 1 (1)  2 (2)  2 (2)  5 (5)  1 (1)  2 (2)  2 (2) |
| **General:**  - flu-like symptoms | 3(*) | 3 (6.5) | / | / | 3 | 3 (3) |
| **Infections:**  - lymph node pain | 2(*) | 1 (2) | / | / | 2 | 1 (1) |
| **Respiratory**:  - allergic rhinitis  - dyspnea  - sore throat | /  1(*)  / | /  1 (2)  / | 1(*)  /  1(*) | 1 (2)  /  1 (2) | 1  1  1 | 1 (1)  1 (1)  1 (1) |
| **Reproductive:**  - dysmenorrhea | 1(*) | 1 (2) | 3(*) | 3 (6) | 4 | 4 (4) |
| **Nervous system**:  - headache  - back pain  - arthralgia  - ankle fracture | 21(*)  1(*)  /  1(*) | 17 (37)  1 (2)  /  1 (2) | 7(*)  /  1(*)  / | 5 (10.6)  /  1 (2)  / | 28  1  1  1 | 22 (24)  1 (1)  1 (1)  1 (1) |

Legend: (1) number of events, (2) number and percentage of subjects, (*) unlikely related to study product, (**) possibly related to study product.

***Table S5: Composition of the L. rhamnosus (test) and placebo product*.**

| **Composition** | ***L. rhamnosus* product (*)** | **Placebo product (*)** |
| --- | --- | --- |
| **Energy** (kcal) | 59 | 60 |
| **Total Sugar** (g) | 6 | 5 |
| Added sugar (lactose) | 1.5 | 0 |
| **Total carbohydrates** (g) | 5 | 5 |
| **Proteins** (g) | 3 | 3 |
| **Total Lipids** (g) | 3 | 3 |
| Saturated fat | 2 | 2 |
| **Lactic acid** (mg) | 650 | <23 |
| **Bacterial strains** (CFU) | 10^11^ (*L. rhamnosus* CNCM I-3690) | / |

Legend: (*) amounts per 100g. CFU, colony-forming units.
